# Benefit and safety of antibiotics for Alzheimer’s disease: Protocol for a systematic review and meta-analysis

**DOI:** 10.1097/MD.0000000000031637

**Published:** 2022-11-25

**Authors:** Lin Gao, Yinqi Shuai, Lihong Wen, Hong Zhang, Yi Zhang, Xiaoyun Zhang

**Affiliations:** a Chengdu University of Traditional Chinese Medicine, Chengdu, Sichuan Province, China; b The Affiliated Hospital of Panzhihua University, Panzhihua, Sichuan Province, China; c Chengdu University of Traditional Chinese Medicine Affiliated Hospital, Chengdu, Sichuan Province, China.

**Keywords:** Alzheimer’s disease, antibiotic, meta-analysis, protocol

## Abstract

Alzheimer's disease (AD) is an age-related degenerative change of the central nervous system, the cause of which remains unclear. Recent studies have found that brain inflammation caused by microbial infections may be one of the etiologies of AD, and antibiotics as novel treatments may be beneficial for delaying the development of AD. Several prospective studies have investigated the effects of different antibiotics on Alzheimer's disease. However, no systematic review or meta-analysis has evaluated the benefits and safety of antibiotics in AD patients.

**Methods:**

This study will analyze randomized controlled trials and observational studies published from database inception to December 31, 2022, and included direct or indirect evidence. Studies will be retrieved by searching PubMed, Scopus, Web of Science, Cochrane Central Register of Controlled Clinical Trials, CNKI, and Wan Fang databases. The outcomes of this study included the Alzheimer's Disease Assessment Scale cognitive subscale (ADAS-cog), Montreal Cognitive Assessment (MoCA), Standardized Mini-Mental State Examination (SMMSE), Clinical Dementia Rating (CDR), Frontal Functioning Scale (FAB), Dysfunctional Behavior Rating Instrument (DBRI), Activities of Daily Living (ADLs) Index, and Geriatric Depression Scale (GDS). The risk of bias will be assessed using the Cochrane risk-of-bias assessment instrument for randomized controlled trials. A random-effect/fixed-effects model will be used to summarize the estimates of the mean difference/risk ratio using a 95% confidence interval.

**Results:**

This study will analyze the benefits and safety of antibiotics in patients with AD.

**Conclusion:**

The results of this analysis will provide evidence to evaluate the benefits and safety of antibiotics in the treatment of AD.

**Strengths and limitations of this study**

This is the first systematic review and meta-analysis to analyze the benefits and safety of antibiotics in patients with AD based on data from both randomized controlled trials (RCTs) and observational studies.

► As is common with most meta-analyses, significant and unexplained heterogeneity may exist.
► The risk for ecological fallacy exists in this study as for any aggregate data meta-analysis.

## 1. Introduction

### 1.1. Rationale

Dementia induced by Alzheimer’s disease (AD) is a global problem that seriously affects the physical and mental health of the elderly population. According to statistics, the number of patients with AD worldwide in 2016 was 43.8 million, an increase of 117% from 1990. By 2050, this figure may increase to 152 million.^[1]^ Behind the huge patient group is the huge medical expenditure. In 2022, the U.S. government is expected to invest $321 billion in health care, long-term care, and hospice care for dementia patients.^[2]^ As the number of patients with AD increases significantly in the coming decades, it is particularly important to understand the pathology of this disease and explore potential treatment methods.

It is generally accepted that extra-neuronal amyloid-beta (Aβ) plaque formation and intraneuronal aggregation of tau proteins in the form of neurofibrillary tangles are two hallmark features of AD.^[3]^ These factors are closely associated with the pathological processes of AD. The production of tau protein is positively correlated with the formation of Aβ plaques,^[4]^ and the lack of clearance of Aβ plaques is also considered the reason for the induction of tau protein to form neurofibrillary tangles.^[5]^ However, Aβ plaque formation is critical for the pathogenesis of AD.^[6]^

Currently, there are limited agents for the treatment of AD. Although cholinesterase inhibitors and memantine play a role in relieving some symptoms of AD, they are not effective in slowing disease progression of the disease.^[7]^ In the past few decades, research on AD treatment has focused on the depletion of Aβ peptides, but the results have not been optimistic.^[7]^ However, patients who received this Aβ peptide-depleting treatment had a higher risk of serious infections, such as meningitis, during clinical studies.^[8–10]^ As studies have found that Aβ deposition occurs 10 to 20 years before clinical symptoms appear,^[11]^ treatments for clearing Aβ plaques seem to be too late for patients. Therefore, targeting the treatment at an earlier stage and preventing excessive formation of the Aβ peptide seems to be a more effective solution.

In recent years, increasing evidence has shown that AD pathogenesis may be related to the invasion of microbes and viruses into the brain, and that the Aβ peptide may be a protective mechanism produced by the innate immune system and exists as an antimicrobial peptide.^[7]^ In fact, researchers have found evidence that pathogens such as viruses, bacteria, and fungi are present in the brains of AD patients.^[12–17]^ Compared with the control group, the number of bacteria in the brains of the patients was 5 to 10 times higher.^[18]^ In contrast, Aβ peptides have been verified to inhibit the proliferation of various bacteria and viruses.^[11,19,20]^ This protective mechanism may be achieved through Aβ peptide oligomerization and trapping. *Staphylococcus aureus* infection can upregulate Aβ expression and aggregation in human tonsils and olfactory fissures, and *S. aureus* aggregates around or is embedded in Aβ deposits.^[21]^ Studies have found that pathogenic microorganisms can directly invade the brain through various pathways, such as the damaged blood-brain barrier, oral cavity, trigeminal nerve, and olfactory system.^[8,22–24]^ Aβ peptides are protein fragments released after the cleavage of amyloid precursor protein on the cell membrane (e.g., neurons).^[25]^ A large number of aβ peptides will aggregate into fibrous networks to capture and aggregate invading microorganisms to limit their proliferation and impact on the surroundings,^[26,27]^ and eventually, these fibrous structures will form insoluble plaques (Aβ plaques).^[25]^

As the infection protection hypothesis is gradually being accepted, an increasing number of researchers have proposed that antibiotics, as a novel treatment method, may be beneficial in delaying the progression of AD.^[7]^ Certain antibiotics have been found to inhibit amyloid formation and promote depolymerization of Aβ42 oligomers.^[28–30]^ Many prospective studies have been conducted on the effects of different antibiotics (including a combination of several antibiotics) on AD.^[31]^ A study combining doxycycline and rifampicin reported improved cognitive function in patients with mild-to-moderate AD using the standardized Alzheimer’s Disease Assessment Scale Cognitive Scale (SADAScog) as the primary outcome.^[32]^ A study on the relationship between *Helicobacter pylori* (HP) and AD found that AD patients who successfully eradicated HP (using a triple eradication regimen including amoxicillin, clarithromycin, and omeprazole) had significantly improved cognitive and functional status parameters compared to AD patients who were infected with HP but did not receive treatment.^[33]^ However, another study noted that the continuous use of doxycycline or rifampicin for 12 months had no significant effect on cognitive function in patients with AD, either alone or in combination.^[34]^ A 2-year study in England and Scotland found that minocycline did not delay the progression of cognitive or functional impairment in patients with mild AD, and that high-dose minocycline was less well tolerated in this population.^[35]^ Not every antibiotic has a positive effect on AD and some antibiotics are harmful. For example, cefepime, an antibiotic that crosses the blood-brain barrier, is known to cause neurotoxic symptoms.^[36]^ Therefore, it is necessary to conduct a systematic review of RCT on the treatment of AD with different types of antibiotics at different dosages and to provide guidance for clinical drug selection.

### 1.2. Objective

The primary objective of this study is to conduct a systematic review and meta-analysis of RCTs and observational studies (OS) to assess the safety and benefit of antibiotics as a component of medical treatment for patients with AD and to develop supporting evidence for effective clinical strategies.

## 2. Methods

### 2.1. Study registration

This protocol is conducted according to the Preferred Reporting Items for Systematic Reviews and Meta-Analyses guidelines^[37]^ or meta-analyses of healthcare interventions. The study protocol follows the Preferred Reporting Items for Systematic Reviews and Meta-Analyses Protocols (PRISMA-P).^[38]^ Supplemental Appendix 1, http://links.lww.com/MD/H864 presents the PRISMA-P Checklist. The protocol has been registered on the PROSPERO network (registration number: CRD42021260042).

### 2.2. Patient and public involvement statement

No patient will be involved.

### 2.3. Eligibility criteria

Studies that meet the following criteria will be included in the analysis: RCTs and OS; patients eligible for trials suffering from AD diagnosed based on the National Institute of Neurological and Communicative Disorders and Stroke/AD and Related Disorders Association classification for probable AD^[39]^ and standardized mini-mental state examination score of 11 to 25 (mild to moderate) inclusive^[40]^; an intervention group will be treated with at least one kind of oral antibiotic; a comparison control group will be included in the randomized trials, which will consist of a placebo, blank, or others; data concerning mortality and other major adverse events will be included; and studies to be included will be published from the database inception to December 31, 2022.

Comparisons will be made between the identified studies that included a placebo or other control compound. RCTs and OS were screened for eligibility. If sufficient RCTs are included, we will conduct a meta-analysis of only the RCTs, and the OSs will appear as supplementary content in the paper. However, if there are fewer high-quality RCTs, we will analyze both RCTs and OSs, and the results of both studies will be analyzed independently rather than mixed.

### 2.4. Information sources

The following databases will be searched from their inception for potentially eligible studies without language restrictions and published up to December 31, 2021: PubMed, Scopus, Web of Science, Cochrane Central Register of Controlled Clinical Trials, CNKI, and Wan Fang. We will also manually search references from relevant RCT identified through systematic reviews, meta-analyses, and studies included in this review.

### 2.5. Search strategy

A systematic search of the 6 public domain databases above mentioned will be performed. The first author will conduct all database searches without language restrictions. The search strategy will be adjusted according to the obtained results and developed using text words and medical subject headings. We will use exploded Medical Subject Headings and appropriate corresponding keywords related to the population, combined with exposure and outcomes such as: “AD” OR “dementia” OR “Cognition disorders” OR “AD” OR “Amyloid beta-Peptides” OR “Senile Plaques” AND “antibiotic” OR “antimicrobial” OR “Anti-Bacterial Agents.” A sample search strategy for PubMed is shown in online supplemental Appendix 2, http://links.lww.com/MD/H865.

### 2.6. Study records

#### 2.6.1. Study selection

**Figure 1.**
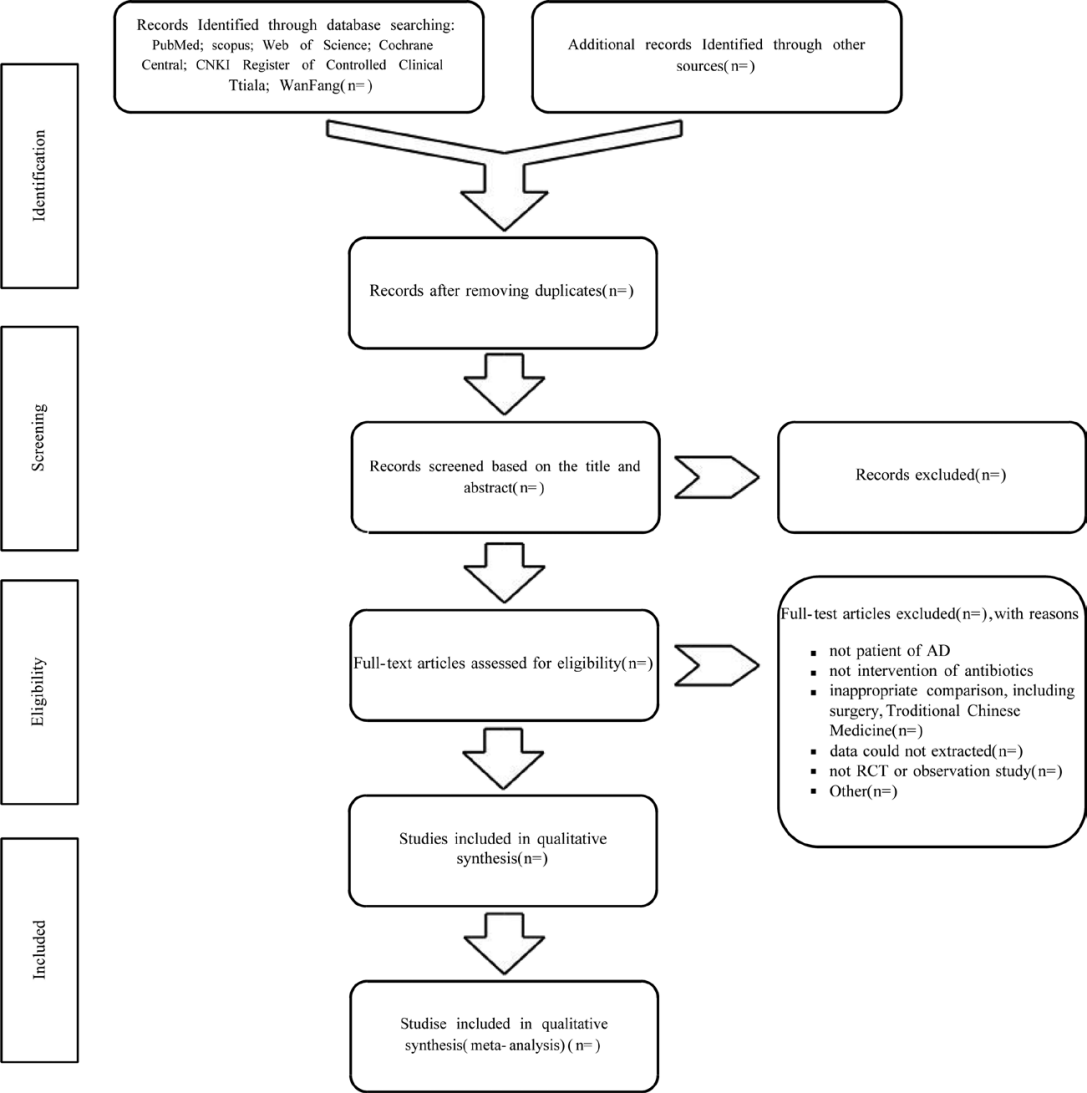
Flow diagram of the study selection process.

All studies will be extracted from electronic databases using the search strategy described above and imported into the Note Express 3.4.0.8878 software (Beijing Aegean Sea Software Company, China). Duplicate studies will be removed. Two authors will select studies independently. Complete articles will be retrieved for all titles and abstracts that appear to meet the eligibility criteria, or where any uncertainty exists. Two reviewers (GL and SYQ) will list all studies to be included and document the primary reasons for excluding studies that did not conform to the eligibility criteria. Disagreements between the two authors will be resolved by discussing with the third author (WLH) and, if necessary, consulting with the fourth author (ZH). The overall agreement rate before correcting for discrepancies will be calculated using Cohen’s kappa (κ). A flow diagram depicting the search process will be constructed. An online supplementary file containing a reference list of all excluded studies, including the reasons (s) for exclusion, will be included in the study. The details of the selection process are presented in the PRISMA flowchart. A flow diagram of the proposed structure is shown in Figure 1.

#### 2.6.2. Data acquisition

Before initiating the data acquisition, a codebook will be developed in Microsoft Excel 2013. Two independent researchers extract data. The following data will be extracted from each eligible study using a standardized data collection form: Study characteristics, including publication year, author, country of the study, type of study (RCT, cohort, case-control, and others), sample size, follow-up duration, and others; participant characteristics, including age, sex, AD type (mild to moderate), and baseline condition of participants (e.g., Alzheimer’s Disease Assessment Scale-cognitive subscale [ADAS-cog], will be used to gauge disease severity); intervention characteristics, including name of the antibiotic(s), dose, route of administration, and others; control characteristics, including the type of drug(s) used, dose, route of administration, and others; and outcome data for ADAS-cog, montreal cognitive assessment, MMSE, clinical dementia rating, frontal functioning scale, dysfunctional behavior rating instrument, ADLs, geriatric depression scale, and adverse reactions caused by antibiotics. The first two authors independently acquired data from the selected studies using the codebook in Microsoft Excel. After data acquisition, both authors reviewed the codebooks and resolved discrepancies by consensus. If a consensus cannot be reached, a third author provides recommendations. A complete list of covariates included in this study is shown in Table 1.

**Table 1:** Covariates that will be included in the study.

Study characteristics: publication year, author, country of the study, type of study (RCT, cohort, case-control, etc), sample size, follow-up duration, others, etc
Participant characteristics: age, sex, Alzheimer’s disease type (mild to moderate), and baseline condition of participants (e.g., Alzheimer’s Disease Assessment Scale-cognitive subscale, [ADAS-cog] will be used to gauge disease severity)
intervention characteristics: name of the antibiotic(s), dose, route of administration, etc;
control characteristics: the type of drug(s) used, dose, route of administration, etc;
Outcome: Alzheimer’s Disease Assessment Scale cognitive subscale (ADAS-cog), Montreal Cognitive Assessment (MoCA), Standardized Mini-Mental State Examination (SMMSE), clinical dementia rating (CDR), frontal functioning scale (FAB), dysfunctional behavior rating instrument (DBRI), activities of daily living (ADLs) Index, and geriatric depression scale (GDS). and adverse reactions caused by antibiotics

### 2.7. Outcomes

The main outcome is the change in AD score from baseline to the last available follow-up, which will be measured using ADAS-cog, montreal cognitive assessment, MMSE, clinical dementia rating, and frontal functioning scale. Additional outcomes include dysfunctional behavior, which will be measured using dysfunctional behavior rating instrument activities of daily living will be measured using the ADL index, and depression will be measured using the geriatric depression scale.

### 2.8. Assessment of the quality of the evidence

According to the grading of recommendations assessment development and evaluation, the quality of the included studies will be assessed using the online guideline development tool (http://gdt.guideline-development.org/) and divided into 4 levels of quality: high, moderate, low, and very low.^[41]^

### 2.9. Risk of bias assessment in individual studies

The risk of bias for RCTs will be assessed using the Cochrane risk of bias instrument,^[42]^ which contains 7 specific domains: random sequence generation (selection bias), allocation concealment (selection bias), blinding of participants and personnel (performance bias), blinding of outcome assessment (detection bias), incomplete outcome data (attrition bias), selective outcome reporting (reporting bias), and other biases. This instrument assesses the methodological quality of RCTs concerning low, high, or unclear risk of bias.^[42]^ The first two authors will independently conduct all risk-of-bias assessments. The two authors then reviewed the results for risk of bias assessment and resolved any discrepancies by consensus. A third author will be consulted if consensus cannot be reached.

The Nine-item Newcastle–Ottawa Quality scale is widely used to assess the quality of non-randomized trials using risk evaluation for the adequacy of selection, comparability, and outcomes assessment.^[43]^ High-quality studies will be defined as those with scores ≥6.

### 2.10. Data synthesis and analysis

#### 2.10.1. Data synthesis

RevMan 5.3 will be used to perform all statistical analyses. For each study, we estimated the standardized mean difference. Standardized mean difference is the mean difference in depression scores between the intervention and control groups divided by the pooled standard deviation (of the distribution of the scores used in the study). The result is a unitless effect-size measure that is readily comparable to other studies using other but similar outcome measures. By convention, effect sizes of 0.2, 0.5, and 0.8 are considered small, medium, and large, respectively.^[44]^ Risk ratios with 95% confidence intervals will be calculated for dichotomous variables.

#### 2.10.2. Assessment of heterogeneity

Statistical heterogeneity among the included studies will be assessed using *χ*^2^ and *I*^2^ tests. Initially, a fixed-effects model will be used for the data analysis. If *I*^2^ > 0.5 or *P* < .1, this will indicate the presence of significant heterogeneity among the studies, and a random-effect model will be used without examining the probable cause for the high heterogeneity.

#### 2.10.3. Subgroup analysis

If there is considerable heterogeneity and the data are sufficient, subgroup analyses will be conducted to identify potential causes of heterogeneity. Subgroup analyses will be performed based on microbial infections, immune system disorders, and the administration of cholinesterase inhibitors and memantine (effective in reducing the progression of AD).^[45,46]^

#### 2.10.4. Assessment of publication bias

Publication bias will be assessed using funnel plots. Additionally, Egger’s and Begg’s tests will be conducted to assess publication bias quantitatively using Stata V. 16.0.

#### 2.10.5. Sensitivity analysis

Sensitivity analyses of the primary results will be conducted to explore the robustness of the review conclusions, if feasible, after considering the impact of methodological quality, missing data, and sample size. According to the Cochrane Handbook, when enough original studies are included (generally >10 trials), publication bias analysis will be performed using a funnel plot. A symmetrical funnel plot indicated a low publication bias. An asymmetric funnel plot indicated a high risk of publication bias.

#### 2.10.6. Software used for data analysis

All data will be analyzed using Stata/IC for Mac V.16.0.

## 3. Registration

In accordance with the Primary Reporting Items for Systematic Reviews and Meta-Analyses Protocols (PRISMA-P), our systematic review and network meta-analysis was registered with the International Prospective Register of Systematic Reviews (PROSPERO) on July 28, 2021 (registration number: CRD42021260042).

## 4. Discussion

Antibiotic therapy may be a novel and promising approach to treating AD. As mentioned earlier, antibiotics can be therapeutically effective in targeting the underlying cause of AD, scavenging pathogenic microorganisms, and preventing the excessive formation of Aβ peptides. However, several issues remain to be addressed. The widespread use of high-dose antibiotics affects intestinal flora.^[47]^ Some preclinical studies have shown that disturbances in gut flora resulting from chronic anti-infective treatment are associated with reduced dementia symptoms in AD as well as reduced Aβ aggregation.^[28]^ However, this finding is inconsistent with the results of other studies. Two months of continuous antibiotic use in middle-aged women can lead to subsequent cognitive decline.^[48]^ It is associated with amyloid aggregation due to dysbiosis of intestinal flora.^[49]^ This suggests that not all patients with AD benefit from antibiotics, particularly those with no clear history of microbial infection. However, dose and regimen are issues that cannot be ignored in the use of antibiotics, and this information becomes unclear in the treatment of AD. One study found that the minimum guaranteed dose of rifampicin to prevent dementia was 450 mg/day for 1 year, which is longer than that of conventional anti-tuberculosis treatment.^[50]^ This means that patients suffer more side effects from antibiotics, which is a major cause of treatment interruption. In a study of different doses of minocycline for AD, a large number of patients withdrew from the study because they could not tolerate the side effects of high-dose minocycline.^[35]^ However, there is no consensus regarding these issues. This study aimed to evaluate the efficacy and safety of antibiotics for AD, including treatment options, severity of dementia, adverse effects, and quality of life. Data on depression, anxiety, and combined medication, if any, will be collected. It will also be determined from the available data whether microbial infections, immune system disorders, use of anticholinergic drugs, etc, affect the outcome of antibiotics in AD. In summary, these analyses provide hypotheses as to which antibiotic regimens may be effective for treating AD-related symptoms. Based on the results of the study, this evidence could form the basis for the design of future clinical trials and development of optimized antibiotic regimens for the treatment of AD.

## Author contributions

GL and SYQ drafted the manuscript. WLH and ZH contributed to the development of data sources to search for relevant literature, including the search strategy, selection criteria, data extraction criteria, and the risk of bias assessment strategy. GL provided statistical expertise, and ZXY and ZY provided content expertise on antibiotics and Alzheimer’s disease. All six authors have read, provided feedback, and approved the final manuscript.

**Conceptualization:** Lin Gao, Lihong Wen.

**Data curation:** Lin Gao, Lihong Wen.

**Formal analysis:** Yinqi Shuai, Lihong Wen.

**Funding acquisition:** Xiaoyun Zhang.

**Investigation:** Yinqi Shuai.

**Methodology:** Yinqi Shuai.

**Project administration:** Hong Zhang.

**Resources:** Hong Zhang.

**Software:** Hong Zhang.

**Supervision:** Yi Zhang, Xiaoyun Zhang.

**Validation:** Yi Zhang, Xiaoyun Zhang.

**Visualization:** Yi Zhang.

**Writing – original draft:** Lin Gao.

**Writing – review & editing:** Lin Gao.

## Supplementary Material
