# Supplementary material for: Benefit and safety of antibiotics for Alzheimer’s disease: Protocol for a systematic review and meta-analysis
Source: Medicine (Baltimore). 2022 Nov 25;101(47):e31637. doi: 10.1097/MD.0000000000031637 (PMC9704868; doi:10.1097/MD.0000000000031637)
Supplement: Supplementary file 2 [file medi-101-e31637-s002.pdf]

PubMed

1. Search: (((((((Alzheimer's Disease[MeSH Major Topic]) OR (dementia[MeSH Major Topic])) OR (Cognition disorders[MeSH Major Topic])) OR (alzheimer*[Title/Abstract])) OR (AD[Title/Abstract])) OR (Amyloid beta-Peptides[Title/Abstract])) OR (Senile Plaques[Title/Abstract])) OR (Aβ[Title/Abstract]) Sort by: Most Recent
2. Search: (((((((((antibiotic[MeSH Major Topic]) OR (Antimicrobial[MeSH Major Topic])) OR (Anti-Bacterial Agents[MeSH Major Topic])) OR (preservative[MeSH Major Topic])) OR (anti-infect*[MeSH Major Topic])) OR (antibiotic*[Title/Abstract])) OR (Antimicro*[Title/Abstract])) OR (Anti-Bacterial Agents[Title/Abstract])) OR (preservative[Title/Abstract])) OR (anti-infect*[Title/Abstract]) Sort by: Most Recent
3. 1 and 2
4. Search: (animals[MeSH Major Topic]) Sort by: Most Recent
5. 3 not 4
6. Search: clinicaltrial[Filter] OR randomizedcontrolledtrial[Filter] Sort by: Most Recent
7. 5 and 6
